# The mycobacterial glycolipid glucose monomycolate induces a memory T cell response comparable to a model protein antigen and no B cell response upon experimental vaccination of cattle

**DOI:** 10.1016/j.vaccine.2009.05.078

**Published:** 2009-07-30

**Authors:** Thi Kim Anh Nguyen, Ad P. Koets, Wiebren J. Santema, Willem van Eden, Victor P.M.G. Rutten, Ildiko Van Rhijn

**Affiliations:** a Department of Infectious Diseases and Immunology, Faculty of Veterinary Medicine, Utrecht University, Yalelaan 1, 3584CL Utrecht, The Netherlands; b Department of Farm Animal Health, Faculty of Veterinary Medicine, Utrecht University, Yalelaan 1, 3584CL Utrecht, The Netherlands; c Department of Veterinary Tropical Diseases, Faculty of Veterinary Science, University of Pretoria, Private Bag X04, Onderstepoort 0110, South Africa

**Keywords:** Glycolipid, Cattle, T cell memory

## Abstract

Glycolipids are presented to T cells by human group 1 CD1 proteins, but are not used as subunit vaccines yet. Experimental immunizations with pure mycobacterial glucose monomycolate (GMM) and keyhole limpet haemocyanin (KLH) in cattle, a species which, unlike mice, expresses group 1 CD1, showed that GMM was equally efficient as KLH in generating T cell responses in blood, but not in the draining lymph node. Also, KLH induced strong antibody responses whereas GMM did not. These data suggest that non-overlapping T cell populations are targeted and demonstrate the potential of glycolipids as a special class of subunit vaccine candidates.

## 1 Introduction

It has been fully established that proteins, including haptenized and glycosylated proteins, are the main targets of the adaptive immune system. More recently, non-protein antigens, including glycolipids, have been shown to be recognized by T cells in vitro. Unlike protein antigens, glycolipids are presented to T cells by the CD1 family of proteins. CD1d presents a limited set of glycolipids to specialized T cells, invariant NKT cells. CD1a, CD1b, and CD1c proteins, collectively called group 1 CD1 proteins, present a more diverse set of lipids to T cells with T cell receptors (TCR) that do not seem to be invariant or otherwise different from regular T cells. Interestingly, not all CD1 isoforms are present in all animal species, which is illustrated by the fact that mice only express CD1d molecules, and no group 1 CD1 molecules. Guinea pigs, cattle, rabbits, pigs, dogs, and humans express at least one group 1 CD1 molecule but the exact numbers and isoforms expressed vary substantially [1–8].

Many of the known lipid-reactive human T cell lines and clones are derived from individuals suffering from a mycobacterial infection, or have been derived in vitro by repeated stimulation of T cells from healthy donors with mycobacterial extracts. This has led to the identification and characterization of a number of CD1-presented mycobacterial antigens including mycolates [9–11], diacylglycerols (LAM) [12], polyisoprenoid lipids [13], sulfotrehalose-containing lipids [14] and lipopeptides [15]. Of note, all mycobacterial antigens appear to be presented by group 1 CD1 (CD1a, CD1b, and CD1c). The mechanism of immune activation by the mycobacterial glycolipid glucose monomycolate (GMM) has been described in great detail [11,16,17]. GMM is known to be presented to human T cells by CD1b. The TCR of the human T cell line LDN5 recognizes GMM of different mycobacterial species that only differ in their lipid tails. The co-crystal of human CD1b with GMM shows that both acyl chains are buried in the antigen binding groove, leaving the glucose moiety exposed on the surface of the CD1 molecule available for recognition by the TCR, explaining why one TCR can recognize GMM from different sources [18].

Using the CD1 system for vaccine development would have the advantage that the molecules it presents are not subject to rapid mutations, like certain antigenic viral proteins. Also, the CD1 system has very limited polymorphism [19], minimizing inter-individual differences in the capacity to present a certain antigen within an outbred population. In addition, CD1-restricted T cells have been shown to be able to express molecules that are very effective in fighting mycobacterial infections [20–22]. Last, many currently used diagnostic tests for mycobacterial infections make use of mycobacterial purified protein derivative (PPD) or related protein preparations. These tests turn positive upon vaccination with BCG or killed bacteria because of priming of T cells that recognize proteins that are present in those vaccines and in the PPD. It is unlikely that immunization with CD1-presented glycolipids interferes with PPD-based diagnostic testing, which would be another advantage of potential lipid-based vaccines.

Even though it has been shown that CD1d-restricted NKT cells support the development of memory B cells, NKT cells themselves express a natural memory phenotype cannot be primed in the sense that immunization with a CD1d-presented antigen improves the strength and the kinetics of subsequent challenges [23–25]. To address the question whether group 1 CD1-restricted T cells can be primed, guinea pigs have been immunized with total lipid extract of *Mycobacterium tuberculosis* [26,27], but no other species have been studied so far. Total lipid extracts are likely to stimulate the CD1 system by several mechanisms, including TLR-mediated adjuvant activity [28], in addition to providing cognate CD1 antigens that are directly presented to T cells. Using pure antigens without known adjuvant activity facilitates the analysis of the adaptive immune response and limits the influence of immune stimulation via mechanisms other than the TCR and/or BCR.

The aim of the current study was to determine whether immunization with a pure mycobacterial glycolipid can prime the adaptive immune system and generate T cell memory, and whether this interferes with the PPD-based diagnostic skin test for tuberculosis. To address these questions, we immunized cattle with the highly purified glycolipid glucose monomycolate (GMM) in the synthetic adjuvant dimethyl dioctadecyl ammonium bromide (DDA). As a control immunogen we used the protein keyhole limpet haemocyanin (KLH) in the same adjuvant. Cattle were chosen for this experiment because cattle are the natural host of several mycobacterial pathogens and therefore a target species for vaccine development. Infections with *Mycobacterium bovis*, causing bovine tuberculosis, and *Mycobacterium avium* ssp. *paratuberculosis*, the causative agent of Johne's disease or bovine paratuberculosis, cause substantial economic losses in the cattle industry [29]. Also, cattle express CD1b molecules [2], which present GMM to T cells from cattle suffering from mycobacterial infection (Van Rhijn et al., manuscript submitted).

## 2 Materials and methods

### 2.1 Bacteria and antigens

*Mycobacterium phlei* and *Nocardia farcinica* were grown in 7H9 culture medium (Difco) supplemented with 10% glucose and 0.5 mg/ml Tween-80 (Sigma–Aldrich). Bacteria were spun down and washed once with distilled water. The wet pellets were extracted for 2 h at room temperature in chloroform:methanol 1:2 and 2:1 (v:v) consecutively. The total lipid extracts were dried in a rotating evaporator at room temperature and redissolved in pure chloroform. For the preparation of GMM, the total lipid extracts were fractionated by loading on a silica solid phase extraction column (Supelco) and consecutive elution with three column volumes of chloroform, followed by three column volumes of 15%, 30%, 40%, 50%, 60%, 70%, and 80% acetone in chloroform, and finally with pure acetone. Quantification and overall analysis was done by thin layer chromatography (TLC) using GMM standards that were analyzed by nanoelectrospray ionization mass spectrometry (ThermoFinnigan LCQ Advantage). After loading the lipids, the TLC plates were resolved in chloroform:methanol:water 60:16:1.5 (v:v) and dried at room temperature. TLC plates were sprayed with 3% cupric acetate in 8% phosphoric acid, dried and baked at 150 °C for 1 h. The fraction containing pure GMM was dried and redissolved in chloroform for storage.

KLH, concanavalin A (conA), and nervonic acid were obtained from Sigma–Aldrich. Phosphatidylinositol, and phosphatidylcholine were from Avanti Lipids.

### 2.2 Animals and immunization

For this study twelve Holstein-Friesian, 2-week-old bull calves were purchased from documented tuberculosis free and paratuberculosis unsuspected dairy herds in The Netherlands. The bulls were group housed and conventionally reared using milk replacer, concentrate and roughage. At the age of 3 months, following a 10-week pre-immunization period, seven animals were immunized subcutaneously with KLH in the left shoulder and with GMM in the other shoulder. Each dose contained either 100 μg GMM or 100 μg KLH (Sigma–Aldrich) in 0.75 ml PBS/5% BSA, and 0.75 ml of a 20 mg/ml suspension of DDA (Sigma–Aldrich) in PBS. GMM was dried under a stream of nitrogen to remove organic solvent and sonicated in PBS/5% BSA. The remaining five animals received two doses of adjuvant only, containing the same components, except for KLH and GMM. A second immunization was performed 1 month after the primary immunization. Two of the GMM/KLH-immunized animals were euthanized at the end of the experiment and their left and right prescapular lymph nodes were collected. Experiments were approved by the Animal Ethical Committee of the University of Utrecht, The Netherlands (protocol numbers DEC 0409.0801 and DEC 2007.II.06.152).

In order to compare humoral responses of animals suffering from an infection with a GMM-producing bacterium and animals exposed to GMM by immunization, sera of animals suffering from clinical paratuberculosis, caused by natural exposure to *M. avium paratuberculosis*, were included in this study. Diagnosis of MAP infection was performed using a faecal culture system [30] at the National Veterinary Health Service, Deventer, The Netherlands.

### 2.3 T cell proliferation assays

T cell assays were performed from 6 weeks before the first immunization till 4.5 months after the second immunization. Peripheral blood mononuclear cells (PBMCs) were isolated from heparinized blood by histopaque ficoll (Sigma–Aldrich) and lymph node cells were isolated by cutting the lymph nodes in small pieces and passage through a cell strainer (BD Falcon). T cell proliferation assays were performed in round bottom 96-well plates (200,000 cells/well). GMM was used in series of dilutions after drying under a stream of nitrogen gas to remove organic solvent and sonicating to be dissolved in T cell medium consisting of RPMI supplemented with 10% FCS (Hyclone), penicillin and streptomycin (Gibco), 20 mM HEPES (Gibco), and 4 ml 1N NaOH. For antibody blocking assays, 20 μg/ml of the anti-human CD1b monoclonal antibody BCD1b.3 or the isotype control P3 was continuously present during the T cell culture. Proliferation was measured after culture for three days in the presence of serial dilutions of KLH, GMM, or concanavalin A (ConA) in a 37 °C, 5% CO_2_ humidified incubator, followed by a 7 h pulse of 1 μCi of [^3^H]thymidine before cells were harvested and counted for β-emissions. Stimulation indices (SI) were calculated by dividing the number of counts per minute obtained with the optimal antigen dilution by the number obtained by incubation with medium alone (Table 1).

### 2.4 Enzyme-linked immunosorbent assays

To assess serological responses, blood was collected in vacutainer tubes (BD), centrifuged, and the serum was collected and stored at −20 °C. Antigen-specific immunoglobulins were measured using enzyme-linked immunosorbent assays (ELISA). Lipids were dried under nitrogen to remove chloroform and sonicated in methanol. Polysorb plates (Nunc, Denmark) were coated with 5 μg/well *N. farcinica* GMM or control lipids (PI, PE, or nervonic acid) and dried overnight at room temperature in a fume hood. Costar high-binding 96-well plates (Corning) were used to coat KLH (0.1 μg/well) by overnight incubation at 4 °C. After blocking with blocking reagent (Roche) for 1 h, 1:30 dilutions of serum in PBS, or PBS only as a negative control, were added to the plates and incubated overnight at 4 °C. Plates coated with GMM were washed with washing buffer consisting of PBS containing/0.05% Tween-20 (Sigma–Aldrich), and plates coated with KLH were washed with washing buffer consisting of PBS/0.25% Tween-20. Biotinylated mouse anti-bovine IgG total (Sigma–Aldrich), diluted 1:50,000 in blocking reagent, was added and incubated for 1 h, followed by three washes with washing buffer, and a 1-h incubation with a 1:4000 dilution of avidine PO (Dako) in blocking reagent. For isotype-specific, antigen-specific ELISA, unlabelled mouse anti-bovine IgG1, IgG2, IgM, or IgA (Prionics), diluted 1:4000 in blocking reagent were added after serum incubation and incubated overnight at 4 °C, followed by three washes with washing buffer and a 1-h incubation with polyclonal rabbit anti-mouse (1:2000) conjugated to HRP (Roche). After three washes with washing buffer and two with PBS, ABTS (Roche) was used to develop green colour which was measured spectrophotometrically at the wavelength of 405 nm. OD values of the wells that were incubated without serum were subtracted from the values obtained with serum.

### 2.5 Skin testing

A single intradermal comparative cervical tuberculin test was conducted according to the regulations (EU directive 64/432/EEC) at the end of the experiment, 4 months after the last immunization. In short, 0.1 ml bovine tuberculin (2000 IU) and 0.1 ml avian tuberculin (2000 IU) (Central Veterinary Institute, Lelystad, The Netherlands) were injected intradermally in the neck of each animal. At 72 h post-injection the skin-fold thickness was measured and corrected for skin-fold thickness measured at time of application. Animals are considered to test positive for *M. bovis* if, after 72 h, the increase in skin thickness at the site of application of bovine tuberculin is more than 4 mm larger than for avian PPD. If the reaction to bovine PPD is between 2 and 4 mm greater than the reaction to avian PPD, this is considered an indeterminate result. Animals showing differences less than 2 mm are considered negative.

### 2.6 Statistics

Following Levene's test for homogeneity of variance, the post-immunization T cell proliferation data and the ELISA data of the two treatment groups were analyzed with one-way analysis of variance (ANOVA) using SPSS 15.0 software. Differences with a *P* value < 0.05 were considered significant.

## 3 Results

### 3.1 Immunization with GMM is as efficient in priming T cell responses as a protein antigen

**Fig. 1:**
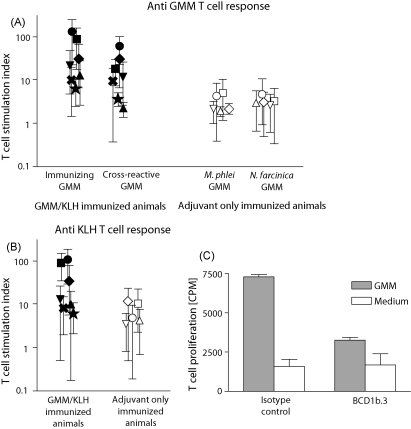
T cell responses against GMM and KLH, induced by immunization. (A) Freshly isolated PBMC of GMM/KLH-immunized animals and adjuvant only immunized animals were stimulated with GMM extracted from *N. farcinica* and *M. phlei*. The cross-reactive T cell stimulation shown is measured by stimulating the T cells with the GMM that was not used for immunization. (B) In the same assay, the T cell response against the protein antigen KLH was determined. The T cell stimulation index was calculated by dividing the [^3^H]thymidine incorporation of the antigen-stimulated PBMC by the [^3^H]thymidine incorporation of PBMC cultured in medium without lipids (Table 1) after a 8-h [^3^H]thymidine pulse performed after a three-day incubation. Each symbol represents the mean stimulation index (±standard deviation) of eight independent experiments performed post-immunization on one animal. Black symbols represent the GMM/KLH-immunized animals and white symbols represent adjuvant only immunized animals. (C) PBMCs of a GMM/KLH-immunized animal were incubated with GMM or medium only in the presence of the cross-reactive anti-human CD1b monoclonal antibody BCD1b.3, or the isotype control P3. Bars represent the mean CPM of triplicate wells (±standard deviation).

To study the efficiency of T cell priming upon immunization with a glycolipid antigen as compared with a known immunogenic model protein, cattle (*n* = 7) were immunized with GMM and KLH in DDA. To reduce the effects of inter-individual differences, while preventing any possible interaction of the two antigens when mixed, the animals received the two antigens separately, in the right and the left shoulder, on the same day. Another group of animals (*n* = 5) of the same age and sex, housed together with the GMM/KLH immunized group, was immunized with adjuvant only and served as a control group. Before the first immunization and from 1 week till 4.5 months after the second immunization, animals were tested every other week for T cell reactivity against GMM and KLH. The GMM specific proliferative T cell response that was detected in freshly isolated PBMC after immunization with GMM/KLH was significantly higher than in adjuvant only immunized animals (*P* < 0.001) (Fig. 1A). A similar pattern was observed for KLH when GMM/KLH immunized and adjuvant only immunized groups were compared (Fig. 1B). Of note, the strength of the response against KLH and against GMM was comparable. Before immunization there were no significant differences between the groups (not shown).

**Fig. 2:**
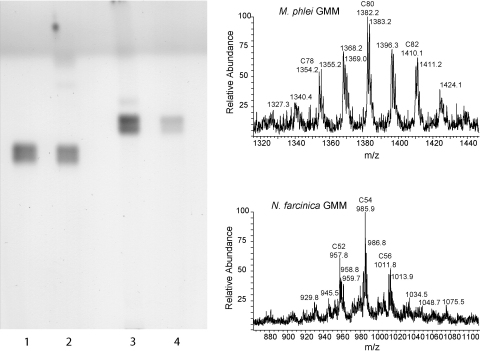
TLC and mass spectrometric analysis of *M. phlei* and *N. farcinica* GMM. Ten micrograms of GMM was applied to a silica TLC plate. The plate was developed in 60:16:2 chloroform:methanol:water (v:v), sprayed with 3% cupric acetate in 8% phosphoric acid, dried, and baked for 1 h at 140 °C. Lane 1: *N. farcinica* GMM standard; lane 2: purified *N. farcinica* GMM; lane 3: *M. phlei* GMM standard; lane 4: purified *M. phlei* GMM. Positive mode spectra were collected by electrospray ionization mass spectrometry.

In order to be able to assess whether a cross-reactive T cell response to GMM would develop, the animals were not immunized with an identical GMM preparation, but rather with GMM from *M. phlei* (*n* = 6) or *N. farcinica* (*n* = 1), which differ in their mycolic acid structure. The purity and the size of the GMM preparations was confirmed by TLC and mass spectrometry (Fig. 2). Cross-reactive T cell responses, measured by using GMM from *N. farcinica* to stimulate T cells from animals that were immunized with *M. phlei* GMM and vice versa, were clearly detected, though they were in general slightly weaker than the response against the GMM that was used for immunization (Fig. 1A). The restriction element for human GMM-specific T cells is CD1b. To confirm whether the GMM-specific T cell response in cattle was restricted by bovine CD1b, we successfully performed antibody blocking assays using the anti-human CD1b antibody BCD1b.3 that has been used for this purpose in the human system, and which is known to cross-react with bovine CD1b [2] (Fig. 1C).

These results indicate that, similar to protein antigen, immunization with GMM antigen raises a T cell response that can be detected in PBMC and that GMM is recognized by T cells regardless of the bacterial source of the GMM.

### 3.2 Immunization with GMM does not cause a positive intradermal skin test for bovine tuberculosis

Four months after the second set of GMM/KLH immunizations, all animals were subjected to the standard comparative intradermal skin test for bovine tuberculosis. Bovine and avian PPDs were applied intradermally and the difference in the increase in skin thickness after 72 h was calculated. In one out of seven GMM/KLH-immunized animals and one out of five adjuvant only immunized animals the increase in skin thickness at the bovine PPD application site was between 2 and 4 mm larger than at the avian PPD application site, qualifying them as indeterminate responders. All other animals were negative.

### 3.3 Unlike KLH, immunization with GMM does not induce a strong antibody response

**Fig. 3:**
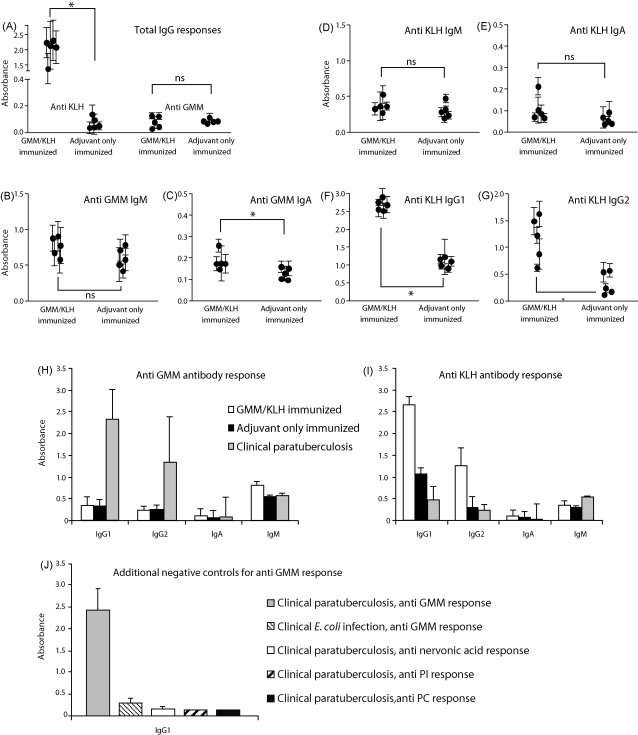
Serological responses of GMM/KLH-immunized animals against GMM and KLH. (A) Sera of GMM/KLH-immunized animals and adjuvant only immunized animals were tested for total IgG responses against KLH and GMM in an ELISA. (B and C) Anti-GMM IgM and IgA were determined using an isotype-specific ELISA. (D–G) The anti-KLH serological response was studied in more detail by determining the individual IgM, IgA, IgG1 and IgG2 responses. Each dot in panel A–G represents the mean absorbance (±standard deviation) of eight independently obtained sera post-immunization from one animal. (H and I) Sera of five animals suffering from advanced paratuberculosis were tested for anti-GMM and anti-KLH antibody responses of the IgM, IgA, IgG1, and IgG2 isotype and compared with the values obtained from the GMM/KLH immunized group and the adjuvant only immunized group. In panel H and I, the anti-GMM IgG1 and IgG2 data as well as the data from the animals suffering from paratuberculosis are based on a single serum per animal; all other data are based on eight independently collected sera per animal. (J) IgG1 responses against GMM, phosphatidylinositol (PI), phosphatidylcholine (PC), and nervonic acid, a C24:1 fatty acid, were determined in sera from five animals suffering from an *E. coli* K88 infection and five animals suffering from advanced paratuberculosis by ELISA.

Antibodies against GMM and KLH in serum were examined by ELISA. Initial testing for antigen-specific total IgG showed that GMM/KLH-immunized animals had generated a strong response to KLH as compared to adjuvant only immunized animals (*P* < 0.01) (Fig. 3A). The situation was different for anti-GMM responses: GMM/KLH-immunized animals as well as adjuvant only immunized animals showed a very weak IgG response against GMM and the difference between groups was not significant (*P* > 0.05) (Fig. 3A). To obtain a more complete view of the antibody responses, ELISAs for antigen specific IgG1, IgG2, IgM, and IgA were performed separately. We detected weak anti-GMM responses of the IgM and IgA isotype (Fig. 3B and C). The IgA response against GMM was significantly higher in GMM/KLH-immunized animals than in adjuvant only immunized animals (*P* < 0.05), whereas the difference in IgM response was not (*P* > 0.05). Also, anti-KLH responses of the IgM and IgA isotype were weak. The differences between GMM/KLH immunized and adjuvant only immunized groups were not statistically significant (*P* > 0.05 for both isotypes) (Fig. 3D and E). High serological responses of the IgG1 and IgG2 isotype were detected against KLH in the GMM/KLH immunized group (*P* < 0.01 for both isotypes) (Fig. 3F and G). Because we did not detect any anti-GMM IgG responses in any of the immunized animals, and no positive control bovine sera with known anti-GMM reactivity were available, we included sera of animals suffering from advanced paratuberculosis in the study. GMM is an immunodominant glycolipid antigen during paratuberculosis, a disease caused by infection of cattle with *M. avium paratuberculosis* (Van Rhijn et al., manuscript submitted). Interestingly, strong anti-GMM IgG1 and IgG2 responses were detected in sera from paratuberculosis infected animals, but no increased IgM and IgA (Fig. 3H). No anti-KLH antibody response of any of the isotypes could be detected in these clinical paratuberculosis sera (Fig. 3I). To rule out the possibility that the anti-GMM response of the sera from paratuberculosis infected animals was reflecting an antigen-independent false positive signal in the ELISA, we showed that the same sera showed no response against the non-relevant lipids phospatidylinositol, phosphatidylcholine, and the long chain fatty acid nervonic acid when tested in parallel with GMM (Fig. 3J). In addition, sera from animals acutely infected with enteropathogenic *Escherichia coli* K88, a bacterium that does not produce GMM, did not show a response against GMM (Fig. 3J).

### 3.4 T cells responses against GMM were not detected in draining lymph nodes, while KLH-specific responses were

**Fig. 4:**
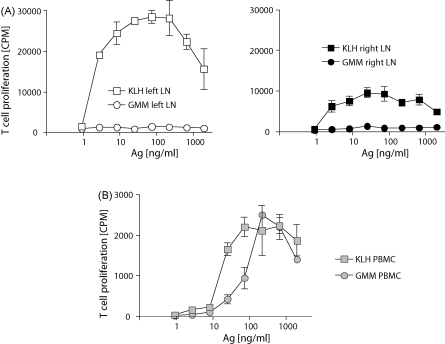
Comparison between T cell responses in draining lymph nodes and PBMC. T cell proliferation assays were performed 4.5 months after the second set of GMM/KLH immunizations using cell suspensions from the left (KLH immunization side) and right (GMM immunization side) prescapular lymph nodes (A), and from PBMC (B). The results shown here were obtained with material from one animal. Highly comparable results were obtained in a second animal (not shown).

Protein-specific T cells can readily be detected in lymph nodes, where they can provide help to B cells, and they are usually highly abundant in the lymph nodes that drain an area of immunization or infection. To assess whether this is also the case for CD1-presented lipid antigen, the left and right prescapular lymph nodes that drained the area of immunization of two GMM/KLH-immunized animals that were euthanized at the end of the experiment were collected and used for T cell proliferation assays. As expected, a very strong anti-KLH response was elicited in both lymph nodes. The response in the left lymph node, where the KLH immunizations were applied, was stronger than in the right lymph node. Interestingly, no anti-GMM T cell response could be detected at all in any of the lymph nodes (Fig. 4A). An experiment performed on the same day on PBMC from the same animals showed clear T cell responses against KLH and GMM of comparable strength, though lower than the KLH responses measured in lymph nodes (Fig. 4B).

## 4 Discussion

Immunization of cattle with a pure glycolipid, GMM, as we describe here induces memory T cell responses but minor antibody responses. Because the GMM that we used was highly purified and did not contain any detectable protein, the observed T cell responses against GMM are probably not caused by protein contaminations present in the GMM preparations. This is supported by the fact that T cells from animals immunized with GMM of one bacterial species recognized GMM from a different bacterial species. So, even if a very low protein contamination would have been present in the GMM preparation used for vaccination, the subsequent in vitro recognition of GMM purified from a different bacterial species cannot be explained by a memory response against protein because even if the preparations contain proteins, they cannot be identical because they would be from different bacterial species.

Even though the anti-GMM T cell response was highly comparable to the T cell response against the control protein KLH in strength and duration, these cells are likely to perform different functions in vivo. Unlike KLH-specific T cells, we showed that T cells that respond to GMM were not detectable in lymph nodes, including the lymph node draining the vaccination site, which is one possible explanation for the big differences in antibody response against these antigens. Another explanation for this is that B cell help is most efficient when the B cell presents the antigen directly to the T cell. In humans and cattle, GMM is presented by CD1b, which is not expressed by B cells. Therefore, cognate B cell help, in the strict sense that the B cell internalizes the antigen that is recognized by its B cell receptor and presents it directly to T cells, cannot take place in the case of GMM. Recently, in an immunization experiment in mice, using a haptenized, CD1d-binding α-galactosyl ceramide analogue, a normal, class-switched antibody response was generated [31]. In this case, cognate B cell help was provided by NKT cells, which was possible because B cells express CD1d. Taken together it seems that, unlike CD1d-restricted NKT cells, CD1b-restricted T cells elicited by immunization with GMM are not capable to provide B cell help. The published in vitro studies are consistent with our observations in the sense that group 1 CD1-restricted T cells are less dependent on fully mature DC with the B7 costimulatory molecules, such as found in lymph nodes, for their activation [28,32] and they express a unique set of effector molecules which is not characteristic for T cells providing B cell help [20,21,33].

The lack of development of antibodies against GMM upon immunization is not due to the absence of a B cell repertoire for this antigen because we have shown that sera of animals suffering from an infection with *M. avium paratuberculosis* do contain antibodies against GMM. A possible explanation for the generation of antibodies during infection and not by immunization may be that during infection in vivo, fragments of bacterial cell walls, that contain other compounds including a variety of proteins, may function like a conjugate vaccine. Also, mycobacteria are known to possess strong adjuvant activity.

The question arises whether the lack of generation of an antibody response will be a major impediment for the use of glycolipids as subunit vaccines. In the case of mycobacteria, it has been suggested that anti-lipid antibodies may provide protection [34,35], but others doubt whether an antibody response provides any protection at all. It is generally accepted that a strong cellular immune response is beneficial for the host. For the protection against other infectious agents, antibodies are required. Antibodies against complex glycosylations can be generated by means of conjugate vaccines, and it might be possible to accomplish the same for glycolipids. Regardless of the question whether the generation of antibody responses against a glycolipid is possible and necessary, it may be worth to use glycolipids in a single or chemically diverse multi-subunit vaccine because group 1 CD1-restricted T cells have some unique features that are not shared by T cells induced by immunization with a regular protein. Thus, a broader range of the immune system will be primed by including a glycolipid in a mixture of antigens. Proteins are known to be able to induce Th1, Th2, cytotoxic, and regulatory T cell responses, but it seems that glycolipids do not induce the same effector T cell populations. We have shown here that glycolipid-specific T cells do not support the generation of strong antibody responses and others have reported that the phenotype of lipid-specific cytotoxic T cell clones does not overlap with peptide-specific cytotoxic T cells [20,21]. Also, it is possible that regulatory T cells can exclusively be found in the peptide-specific, MHC class II-restricted T cell population, and if so, immunization with pure glycolipids opens a possibility to avoid the stimulation of regulatory T cells.

We have shown that immunization with GMM does not cause the standard intradermal tuberculosis test in bovines to turn positive. This is likely due to the absence of lipids and glycolipids in purified protein derivative (PPD). If it appears in the future that vaccines exclusively consisting of lipids and/or glycolipids provide a reasonable level of protection against mycobacterial infectious diseases, a major advantage of using them, instead of the currently used vaccines based on attenuated mycobacteria, like *M. bovis* BCG, which provides some protection against tuberculosis, or killed mycobacteria, like the Gudair vaccine against bovine paratuberculosis, is that they do not cross-react with PPD-based diagnostic tests.

## Figures and Tables

**Table 1:** Proliferation data, expressed in counts per minute (CPM), that form the basis of the stimulation indices presented in Fig. 1.

Animal#	Immunization	Symbol in Fig. 1	CPM GMM^a^	SD	CPM KLH	SD	CPM medium	SD	CPM ConA	SD
0041	*M. phlei* GMM/KLH	■	19,838	19,581	22,300	24,425	320	538	185,909	94,281
0472	*M. phlei* GMM/KLH	●	18,863	22,569	16,863	12,853	204	1120	167,182	83,289
2629	*M. phlei* GMM/KLH	▾	4,233	1,411	2,687	1,588	278	289	205,727	43,388
3051	*M. phlei* GMM/KLH	✖	2,660	2,542	2,665	2,301	357	359	240,182	55,587
4815	*N. farcinica* GMM/KLH	★	1,340	1,149	1,410	1,267	267	232	138,136	96,760
5083	*M. phlei* GMM/KLH	♦	9,603	7,198	9,338	5,663	467	638	169,818	66,562
9596	*M. phlei* GMM/KLH	▴	4,915	1,799	4,413	2,388	595	758	237,364	59,488
0177	Adjuvant only	▵	451	419	599	425	197	186	171,545	75,306
3050	Adjuvant only	▿	2,190	1,996	1,519	691	803	598	210,545	54,959
4014	Adjuvant only	◇	668	376	2,206	784	290	256	244,273	73,157
6597	Adjuvant only	○	2,605	2,497	1,913	816	725	630	199,636	43,058
7408	Adjuvant only	□	2,026	1,395	4,491	3,007	526	764	203,636	54,397

a Average of CPM obtained upon in vitro stimulation of PBMC with the same GMM that was used for immunization, or with *M. phlei* GMM for stimulation of PBMC of adjuvant only immunized animals.
